# Design of Ascorbic Acid Eutectic Mixtures With Sugars to Inhibit Oxidative Degradation

**DOI:** 10.3389/fchem.2022.754269

**Published:** 2022-05-09

**Authors:** Vasanthi Palanisamy, Palash Sanphui, Kandhan Palanisamy, Muthuramalingam Prakash, Arvind Kumar Bansal

**Affiliations:** ^1^ Department of Chemistry, Faculty of Engineering and Technology, SRM Institute of Science and Technology, Chennai, India; ^2^ Department of Pharmaceutics, National Institute of Pharmaceutical Education and Research (NIPER), Mohali, India

**Keywords:** ascorbic acid, sugar, eutectics, lamellar microstructure, shelf-life, binding energy

## Abstract

L-Ascorbic acid (ASC), commonly known as vitamin C, acts as an anti-oxidant in the biological system. It is extensively used as an excipient in pharmaceutical industry, food supplements in fruit juices, and food materials due to its free radicals scavenging activity. Main drawback of ASC is its poor aqueous stability owing to the presence of lactone moiety that is easily oxidized to dehydroascorbic acid and further degraded. To improve aqueous stability and inhibit oxidative degradation, ASC was co-crystallized to constitute binary eutectic compositions with mono and di-saccharides such as glucose, sucrose, lactose, and mannitol. The eutectics were confirmed by their (single) lower melting endotherm compared to ASC and sugars, although Powder X-ray diffraction (PXRD) and Fourier transform Infrared spectroscopy (FT-IR) data confirmed the characteristics of their physical mixture. Scanning electron microscope (SEM) images of the binary eutectics confirmed their irregular morphology. The ASC eutectics exhibited improved shelf-life by 2–5-fold in weakly acidic (pH 5) and neutral (pH 7) aqueous buffer medium, whereas the eutectic with glucose enhanced shelf-life only by 1.1–1.2-fold in acidic medium (pH 3.3 and 4). Notably, stabilizing effect of the sugar eutectics decreased with increasing acidity of the medium. In addition, higher binding energy of the disaccharide eutectics partially supports the aqueous stability order of ASC in the neutral pH medium due to more number of non-bonded interactions than that of monosaccharides.

## Introduction

Importance of crystalline solid forms (e.g., polymorphs, cocrystals, salts, solid solutions, eutectics) in altering pharmaceutically relevant properties like solubility, bioavailability, stability, and tableting is well known in the prior art (Sarma et al., 2011; Cherukuvada and Nangia, 2014; Couillaud et al., 2019). Eutectics are homogeneous at a fixed stoichiometry and held by non-bonded interactions between two or more component systems (Rajbongshi et al., 2018; Sathisaran et al., 2018; Jagia et al., 2019; Saeed et al., 2021). They are characterized by lower melting points compared to the individual components, when spectroscopic tools prove them as physical mixtures. The literature related to the organic eutectics is limited compared to their cocrystal counterparts due to difficulty in characterizations and understanding their structural aspects. Pair distribution function analysis often suggests the local structural domain in the lattice of eutectic microstructure (Chirawatkul et al., 2019). The most anticipated lamellar microstructure of eutectics may adhere *via* weak Coulombic forces. Enhanced wettability, reduced particle size, and microcrystalline structure of the eutectics often contribute to the improved drug properties (Bazzo et al., 2020).

L-ascorbic acid (ASC) consists of asymmetric six carbon atoms and is structurally related to glucose. It exists as colorless solid that usually turns yellowish during solid-state degradation. Among two enantiomers (D/L), the l-isomer is predominantly found in fruits (e.g., strawberry, citrus fruits), vegetables (e.g., broccoli, capsicum), and sold as a dietary supplement. It plays an important role in collagen synthesis and as an antioxidant (radical scavenger) in the biological system (Bendich et al., 1986; Colven and Pinnell, 1996). ASC is an essential nutrient required in the repair of tissue and enzymatic synthesis of specific neurotransmitters (Dietary reference, 2000). It is well tolerated and a maximum dose of 500 mg is generally prescribed. ASC has been extensively used in the pharmaceutical, chemical, cosmetic, and food industry due to its bioactivity and antioxidant properties. Lactone moiety in ASC makes it vulnerable to acid/base hydrolysis. Besides, the ene-diol system of ASC easily donates electrons to form l-dehydroascorbic acid in the aqueous phase. ASC is a weak acid (*p*Ka = 4.2), and dissociated into ascorbate form with increasing pH of the aqueous medium (Kimoto et al., 1993).

In biological systems, ASC can be found only at low pH (Kimoto et al., 1993; Dietary reference, 2000) and the ionized form exists predominantly in solutions above pH 5. In an acidic medium, ASC degrades to 2-furoic acid, 3-hydroxy-2-pyrone, and furfural *via* dehydroascorbic acid (Yuan and Chen, 1998). It degrades to furfural and other unknown products in an alkaline medium. The main degradation product of ASC is dehydroascorbic acid, which is metabolized to furfural, 2-furoic acid, 3-hydroxy-2-pyrone, etc. Degradation of ASC is affected by several factors like light, pH and viscosity of the medium, dissolved oxygen, metal ions (e.g., Cu^2+^/Fe^3+^), and temperature (Robertson and Samaniego, 1986; Muller, 2001; Burdurlu et al., 2006; Wechtersbach and Cigić, 2007; Sapei and Hwa, 2014). Rate of ASC degradation in the aqueous phase is initially prompt during storage due to instantaneous reaction with the dissolved oxygen, followed by gradual degradation (Verbeyst et al., 2013). Commercial fruit juices with added ASC (as an additive) develop degraded products like furfural and 2-furoic acid during prolonged storage conditions (Tikekar et al., 2011).

Extensive research has been carried out to stabilize ASC either in the aqueous phase or molten/slurry phase. Ethylenediaminetetraacetic acid is reported to stabilize ASC for 2 h in an acetate (pH 6) buffer medium (Salkić and Selimović, 2015). A fused molten mixture of ASC and mannitol (1:9, w/w) exhibited rapid congealing properties than their eutectic mixture (2:3, w/w) (Osugi et al., 1968). Addition of stabilizers (5–10%) like glutathione, ferulic acid, citric acid, stearic acid, tartaric acid, sodium metabisulphite, ubiquinone, glucose, and sucrose are reported to increase shelf-life of ASC (Sonni et al., 2011; Ahmad et al., 2012; Corvis et al., 2013; Ahmad et al., 2015). Tikekar et al. examined UV light induced ASC degradation in apple juice in the presence of 10% carbohydrates (Tikekar et al., 2011). Topical formulation of ASC was stabilized by several emulsions with α-tocopherol, boric acid, citric acid, and tartaric acid (Rozman and Gasperlin, 2006; Ahmad et al., 2015). Ferulic acid stabilized aqueous solution of 15% ASC and 1% Vit-E improved 8-fold photoprotection for skin to solar simulated irradiation (Lin et al., 2005). A deep eutectic solvent of ASC and choline chloride (1:2) maintained its antioxidant properties up to 6 months (Silva et al., 2018). An ASC solution in normal saline with 5% dextrose was photostable at freezer condition for at least 14 days (Walker et al., 2019).

In spite of large database on the stability profile of ASC, no quantitative data of the effect of stoichiometric amount of coformers are available on the aqueous stability of ASC in the literature. Even, prior art did not discuss the appropriate reason behind their stabilizing behavior or role of these additives in stabilizing ASC in aqueous medium. Moreover, multicomponent systems like cocrystals, salts, and eutectics of ASC have not been exploited to prohibit oxidative degradation in the aqueous phase. Cocrystals are well known in the prior art to improve chemical stability of a few active ingredients (Babu et al., 2012; Battini et al., 2018), whereas literature related to enhanced stability by eutectics are rarely found (Cherukuvada and Nangia, 2014).

Crystal structures of ASC and its cocrystals with sarcosine, betaine, l-serine, nicotinic acid, picolinic acid, isoquinoline carboxylic acid, nicotinamide, isonicotinamide, and salt with arginine are reported in the literature (Sudhakar et al., 1980; Sudhakar and Vijayan, 1980; Goher et al., 2003; Kavuru et al., 2010; Wang et al., 2016; Evtushenko et al., 2020). Most of the cocrystal structures consist of hydrogen bonding between ene-diol of ASC with the coformers. Although, none of the cocrystals have addressed the degradation problems of ASC in the aqueous phase, the oxidative degradation occurs *via* the attack of water or hydroxyl group (alkaline medium) to the lactone moiety and transformation into dehydroascorbic acid followed by ring opening reaction (Yuan and Chen, 1998). Hence to stabilize ASC in the aqueous phase, if the ene-diol and lactone fractions can be encapsulated with the coformers *via* noncovalent interactions like hydrogen bonding, Coulombic interactions, degradation may be inhibited.

**SCHEME 1:**
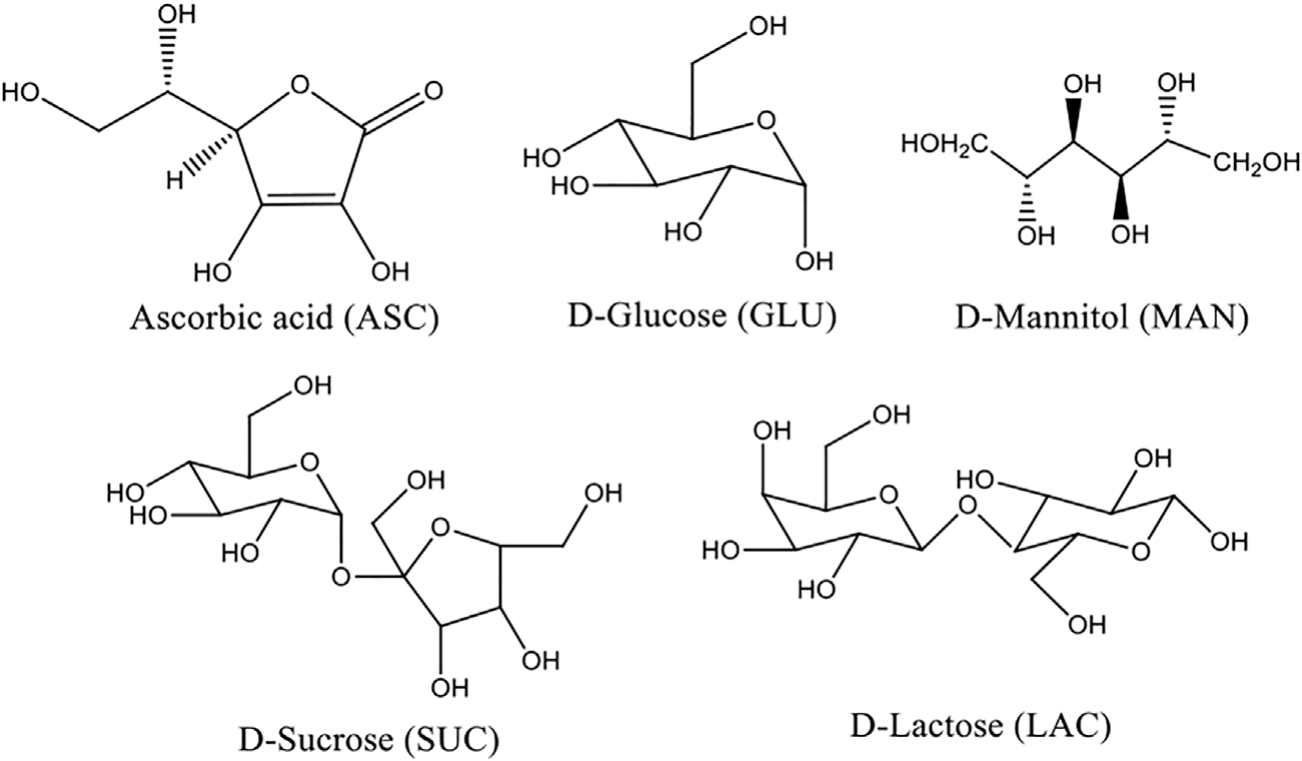
Chemical diagrams of ASC and its eutectic coformers.

**SCHEME 2:**
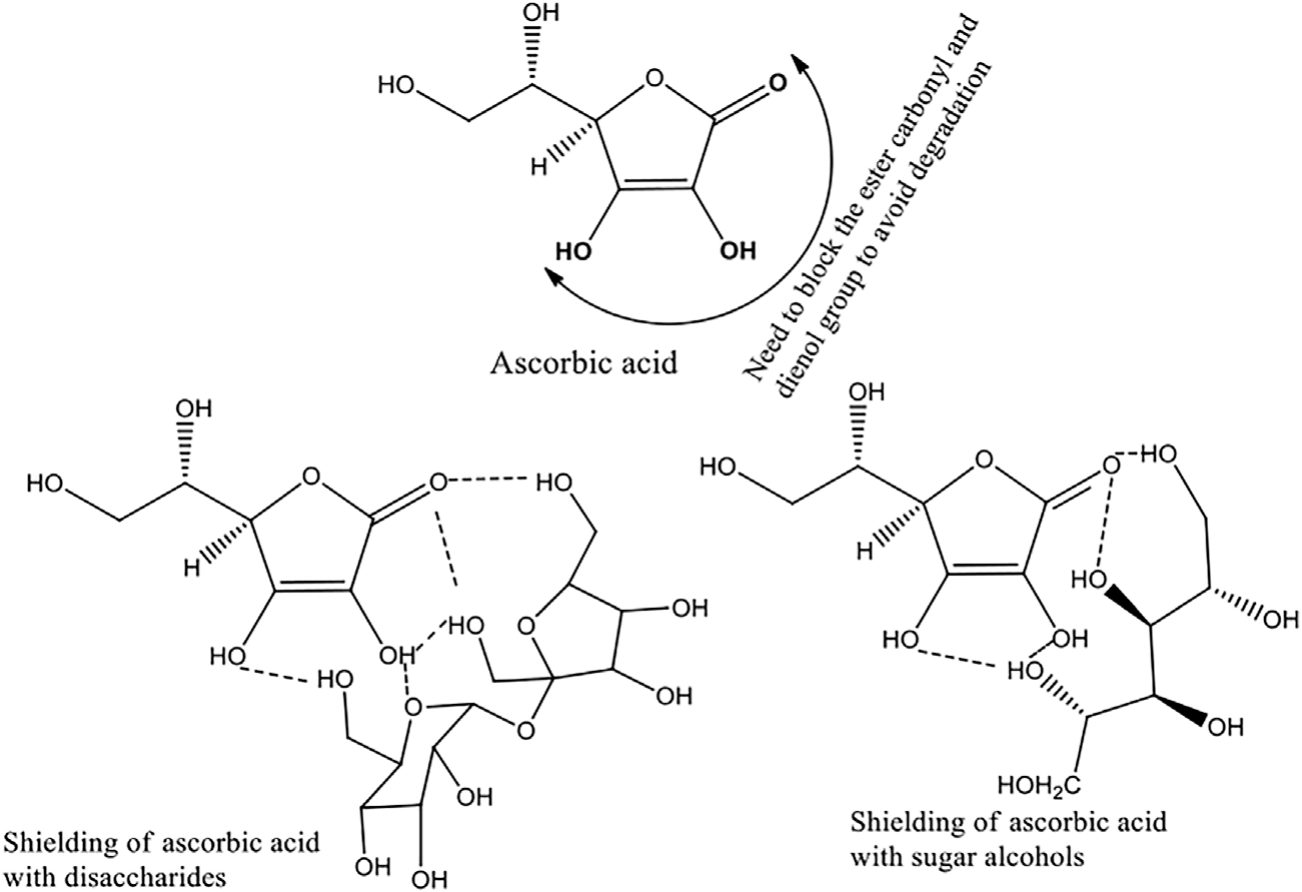
Proposed non-bonded interactions in ASC eutectics with sugars and sugar alcohols to inhibit oxidative degradation.

In this context, sugar moieties like d-glucose, d-sucrose, d-lactose, and mannitol were cocrystallized with ASC (Scheme 1). Sugars with weakly acidic (SP^3^ hybridized carbons) hydroxyl groups form eutectics with ASC that may block the degradation pathways *via* shielding using auxiliary interactions (Scheme 2). Wet granulation of ASC with the sugars in an equivalent stoichiometry resulted in novel binary eutectic mixtures due to lack of synthon compatibility. XRD and Fourier transform Infrared spectroscopy (FT-IR) data concluded the ground binary ASC adduct as physical mixture. Although, differential scanning calorimetry (DSC) confirmed them as novel eutectic solid phases, the degradation of ASC is very slow and almost comparable in pH 1–3 medium and rapidly increases beyond pH 4.2 due to ionization to dehydroascorbic acid. The aqueous stability of the binary eutectics was implemented in pH 3.3, 4, 5 (acetate), and 7 (phosphate) buffer medium at 25°C to examine the effect of an equivalent eutectic (sugar) coformers to inhibit the ASC degradation. In addition, stabilizing effect of the binary eutectics was partially correlated with their computed binding energy (BE).

## Experimental Section

ASC was procured from Sigma Aldrich, Bangalore, and other coformers (d-glucose, d-sucrose, d-lactose monohydrate, mannitol, nicotinic acid, nicotinamide, l-serine and sarcosine) were obtained from SRL, Chennai. ASC and coformers (>98% purity) were used without further purification in this study. Methanol used as lubricant during eutectic preparations was of analytical grade. Milli Q water was used to prepare the different buffer media.

## Methods of Preparation of Binary Eutectics

The ASC binary eutectics with sugars were prepared according to the common procedure. ASC (1 mmol) and sugar (1 mmol) were subjected to grinding using a mortar and pestle. The grinding was continued for 15 min by adding a few drops of methanol at regular intervals of time. The ground mixture was dried under vacuum at ambient conditions. Note, the ASC cocrystals with nicotinic acid, nicotinamide, l-serine, and sarcosine were prepared according to the literature procedure (Kavuru et al., 2010; Wang et al., 2016).

### Powder X-Ray Diffraction

A PANalytical X’Pert Pro powder X-ray diffractometer was employed to record the XRD patterns of the ground ASC binary mixtures, ASC, and sugar coformers during multicomponent solid form screening. Data collection was carried out at room temperature using Cu *K*ɑ radiation (1.5418 Å; 40 kV, 30 mA) as the X-ray source in 2θ continuous scan mode (Bragg–Brentano geometry) in the range 5–50° at a scan rate of 1° min^-1^ and a time per step of 0.5 s.

### Infrared (IR) Spectroscopy

A Perkin Elmer IR spectrometer was used to record the vibrational frequencies of the ground ASC-sugar binary mixture, ASC, and sugar coformers. IR spectra were recorded on the powder samples in the ATR (Attenuated Total Reflectance) mode.

### Thermal Analysis

DSC of the ground ASC binary mixtures and ASC were performed on a Diamond DSC PerkinElmer-10 module. About 2–3 mg of each sample was placed in a crimped and vented aluminum sample pan and heated at a rate of 10 °C min^-1^ up to 300°C under a dry N_2_ atmosphere. The enthalpy of fusions was calculated from the integration of the area of the melting peak and the interpolated baseline between the beginning and end of the endotherm. A Perkin Elmer Pyris-1 TGA (Thermogravimetric analysis) instrument was employed by heating 10 mg sample of ASC-LAC (hydrate) in an open aluminum crucible in the temperature range of 30–500°C at 10°/min.

### Particle Morphology

Particle morphology of the ASC binary eutectics was examined using a field emission scanning electron microscope (FEI, Quanta 200) operated at an excitation voltage of 15 KV. Fine powders of the eutectics were mounted onto steel stage using double-sided adhesive tape and coated with gold using ion sputter.

### Degradation Study

The degradation of ASC and its binary eutectics were carried out in pH 3.3, 4, 5 (acetate), and pH 7 (phosphate) buffer medium at λ_max_ of 245–265 nm on a standby mode using Cary 6000i UV-Vis-NIR Spectrophotometer (Agilent Technologies). In addition, malic acid solution (0.5% w/v) as model apple juice was used to carry out degradation study of ASC. About 2 mg ASC (0.011 mmol) and an equivalent eutectics were dissolved in freshly prepared buffer medium at 25°C and diluted accordingly (final conc. ∼12.5 mg/l) to measure the absorbance. The aqueous stability was monitored by measuring absorbance at a certain stipulated time interval, preferably 5–10 min. The degradation of ASC was calculated based on its half-life in the buffer medium.

### Computational Details

Density functional theory (DFT) calculations were carried out by using Gaussian 16 program (Frisch et al., 2016). All the selected models and the different isomers of ASC heterodimer with glucose, mannitol, sucrose, and lactose monohydrate were optimized by using M05–2X/6–311++g (d, *p*) method. The M05–2X functional is more suitable for non-covalent interactions, i.e., H-bonding and van der Waals (Zhao et al., 2006). Frequency calculations were performed at the same level of theory to confirm the minima of the geometries, where the lowest energy structures were considered as the global minima in the potential energy surface. The effect of solvation was studied by using SCRF-PCM (water) approaches. The BE of all the complexes was calculated by using the counterpoise method, to avoid the basis set superposition error (BSSE) (Boys and Bernardi, 1970).

$$BEs=\;-\left[E_{Cluster}-\left(E_{ASC}+E_{Sug}\right)\right]$$ (1)

where E_Cluster_, E_ASC_, and E_Sug_ are the total energies of complex, ASC, and sugars, respectively. Further to study the effect of dispersion energies in the complexes, BEs were calculated by Grimme D3 dispersion corrections along with the counterpoise method. Atoms in Molecules (AIM) analysis, the electron [ρ(r_c_)], and Laplacian density [∇^2^ρ(r_c_)] were calculated by using AIM2000 package (Biegler-Konig et al., 2001).

## Results and Discussion

**FIGURE 1:**
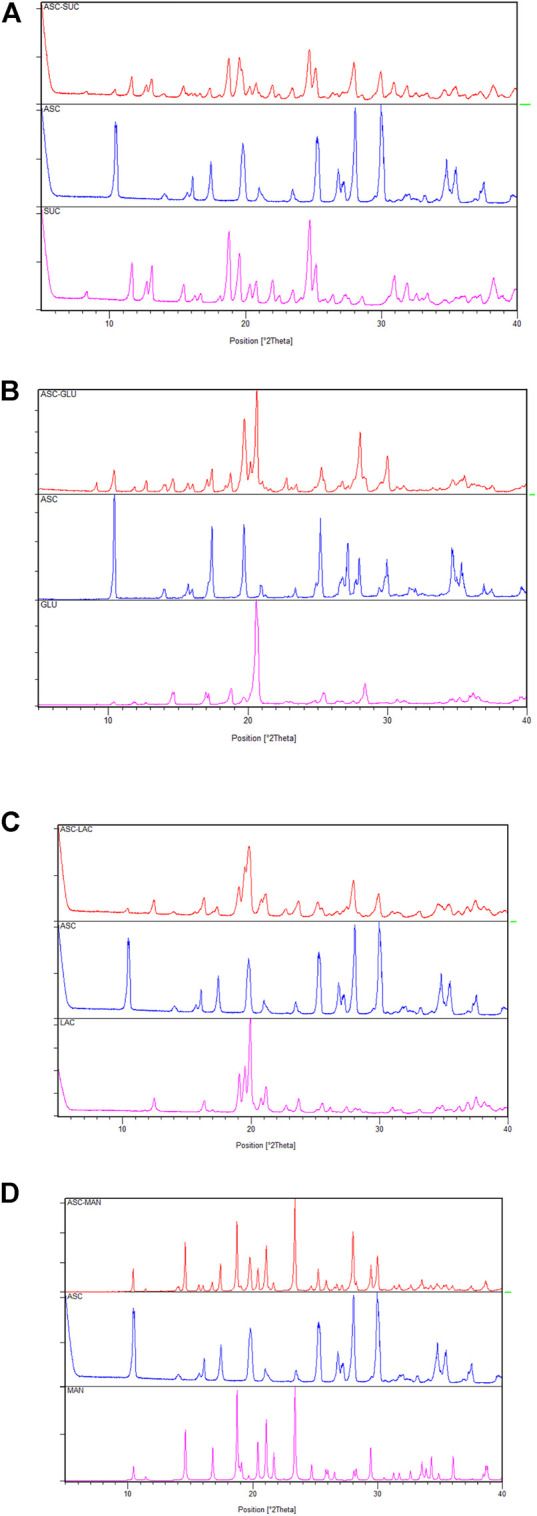
Powder X-ray diffraction (PXRD) comparison **(A–D)** of the ascorbic acid + sugar (ground mixture) with the ASC and coformers indicate physical mixture of individual components and there is no strong hydrogen bonding interactions between them.

The ASC and sugar binary ground mixtures were characterized by XRD and Field Emission Scanning Electron Microscope. The liquid assisted an equivalent ground mixture of ASC and sugars exhibited the diffractions patterns, which are superimposition of the characteristic peaks of individual compounds, see Figure 1 and Supplementary Figure S1, The XRD pattern of ASC at 10.4, 17.5, 25.3, 28.0, 30.0 ± 0.2 2θ was preserved in the ASC binary compositions. Intensity of the XRD patterns of ASC-SUC varied that could be due to smaller particle size formation, which are well-known in the literature (Ganduri et al., 2017; Chaturvedi et al., 2020). Similarly, the vibrational frequencies of ground mixture of ASC and sugars are very close with their individual counterparts. Positions of C=O vibrations at 1655, 1754 cm^‒1^ and O-H stretching at 3527, 3406, 3313, and 3212 cm^‒1^ are mostly retained in the ASC-sugar ground mixtures (Supplementary Figure S2). More prominently, all the ASC-sugar ground mixture showed much broader peaks in the O-H stretching range due to more number of hydroxyl groups involved in non-bonded interactions. Both the XRD and FT-IR spectra confirmed the ground adduct of ASC and sugar compositions as physical mixture and there is no strong hydrogen bonding interactions between them.

**FIGURE 2:**
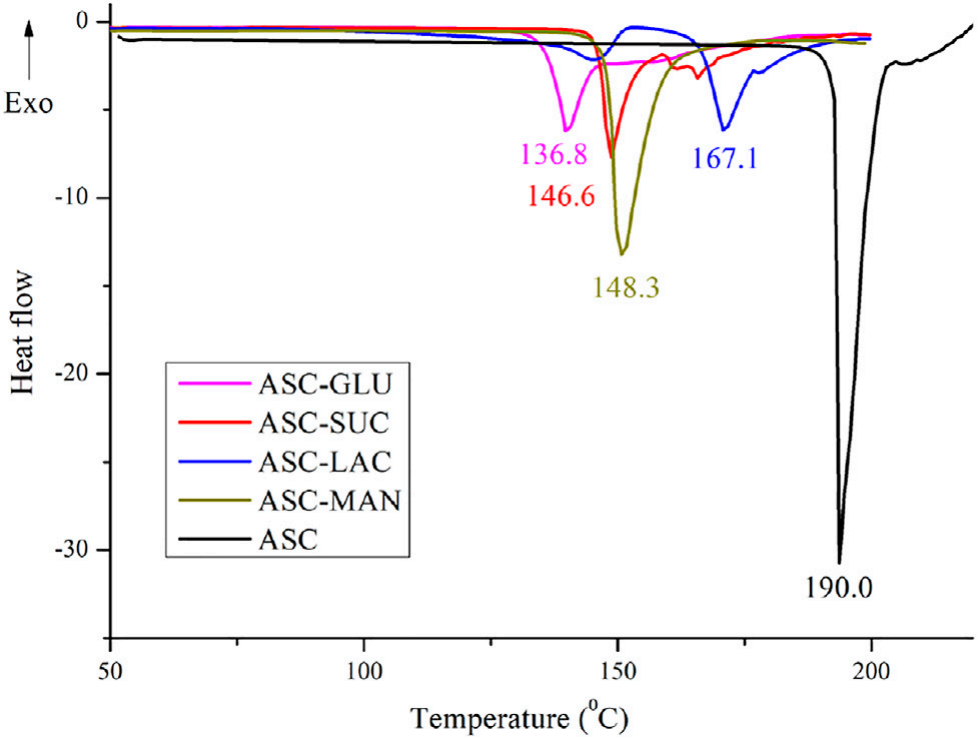
DSC heating curves of ASC-sugar (1:1) eutectics confirmed single phase entity.

All the binary solids were confirmed as the eutectic mixtures by their lower melting points compared to the individual components as their formation is entropy driven process. This indicates possibility of weak electrostatic interactions between two components. Non-compatible heterosynthon between hydroxyl groups of sugar and carbonyl group of ASC results in the formation of eutectic. In addition, similar crystal structure of ASC and sugars also play an important role in dictating eutectic compositions between them. If the binary solids are simple physical mixture, two endotherms corresponding to individual component could be observed. DSC heating curves of binary eutectics are displayed in Figure 2. All the eutectics (except ASC-LAC) are anhydrous phases, which showed lower melting endotherm compared to ASC and sugar coformers. ASC-LAC (hydrate) showed broad endotherm of dehydration temperature at 133–146°C, followed by melting at 171°C. Note, the eutectic coformer, lactose monohydrate (LAC) exhibited dehydration at 145–148°C (sharp endotherm) and melting at 217°C. The difference in dehydration pattern of LAC (hydrate) and ASC-LAC (hydrate) suggests that the water molecule plays an important role in ASC-LAC eutectic formation, see Supplementary Figure S3. In addition, TGA of ASC-LAC indicated the dehydration started at 115–120 °C with a weight loss of 4%, which is little higher than the calculated weight loss (3.3%) for one equivalent of water. Melting points, along with the enthalpy of fusion of ASC eutectics, are summarized in Table 1. When the melting point of the two eutectic coformers is close, there is a high probability of formation of the (1:1) eutectic mixture, which partially supports the melting endotherms of ASC-sugar eutectics with an equivalent stoichiometry (Vippagunta et al., 2007). According to Jackson and Hunt proposal, on the correlation between entropy of fusion (ΔS_f_) of individual eutectic coformers and microstructure of eutectics (Hunt and Jackson, 1966), both ASC and sugar coformers possess ΔS_f_ <2, see Table 1. It can be concluded that both the ASC and sugar phases grow together behind a planar solid-liquid interface to exhibit a well-defined successive lamellae or rod-like microstructure of one-phase encapsulated in another.

**TABLE 1:** Melting points (DSC data) of the ASC eutectics.

ASC/ASC-Eutectics	M. p. (°C) of ASC/ASC Eutectics	Enthalpy of Fusion (J/g), ΔH_f_	Entropy of Fusion (J/g/K), ΔS_f_	M. p. (°C) of Coformers
ASC	190–192.8	−902.2	1.94	--
ASC-GLU (1:1)	136.8–140.0	−146.3	0.35	146
ASC-SUC (1:1)	146.6–148.4	−120	0.28	186
ASC-LAC (1:1)	167.1–171.2	−143.2	0.32	214
ASC-MAN (1:1)	148.3–151.1	−469.5	1.11	170

Microstructure characterization.

The ASC crystallized as square plate crystals in methanol. But, the eutectics of ASC and sugars got separated during crystallization attempt and predominantly crystallized as ASC crystals. Hence to gain insight of the ground eutectic samples, powder samples of the ASC eutectics were developed on a glass slide and visualized under using Field Emission Scanning Electron Microscope (FESEM), which confirmed irregular particle morphology, see Supplementary Figure S4. The particles were smaller in sizes in the range of 2–5 μm that is common phenomenon among the eutectic phases (Chaturvedi et al., 2020). As the ground particles did not adopt a suitable geometry, it was difficult to measure their exact dimensions.

### Aqueous Stability Study

**FIGURE 3:**
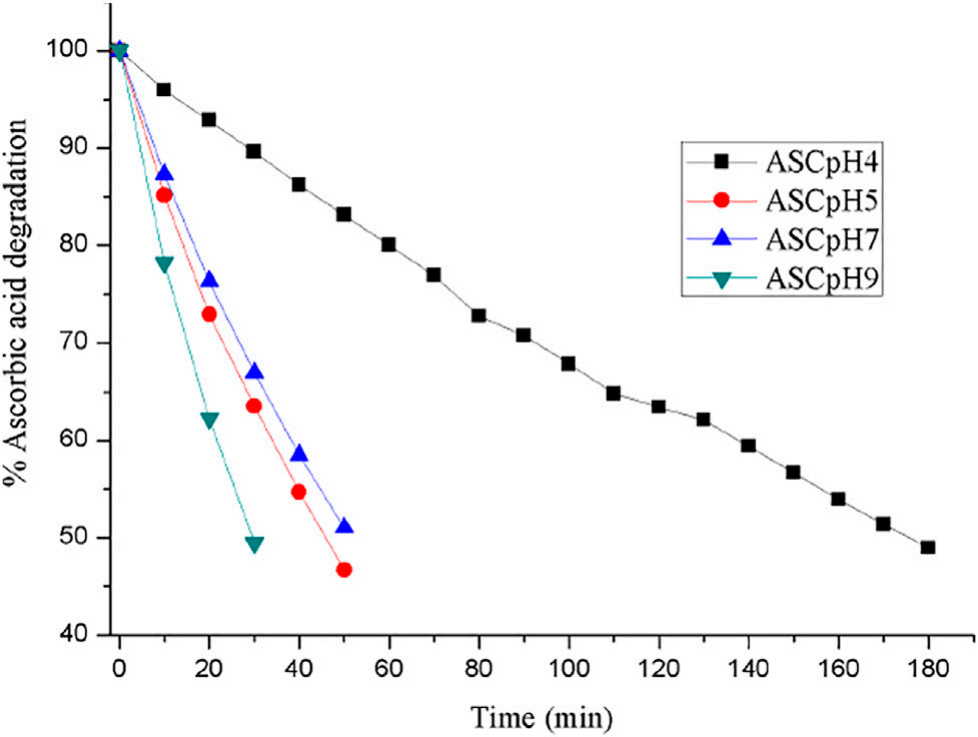
ASC degradation with increasing pH (4→9) that reflects its shorter half-life (UV measurements are recorded at 5 min interval).

**FIGURE 4:**
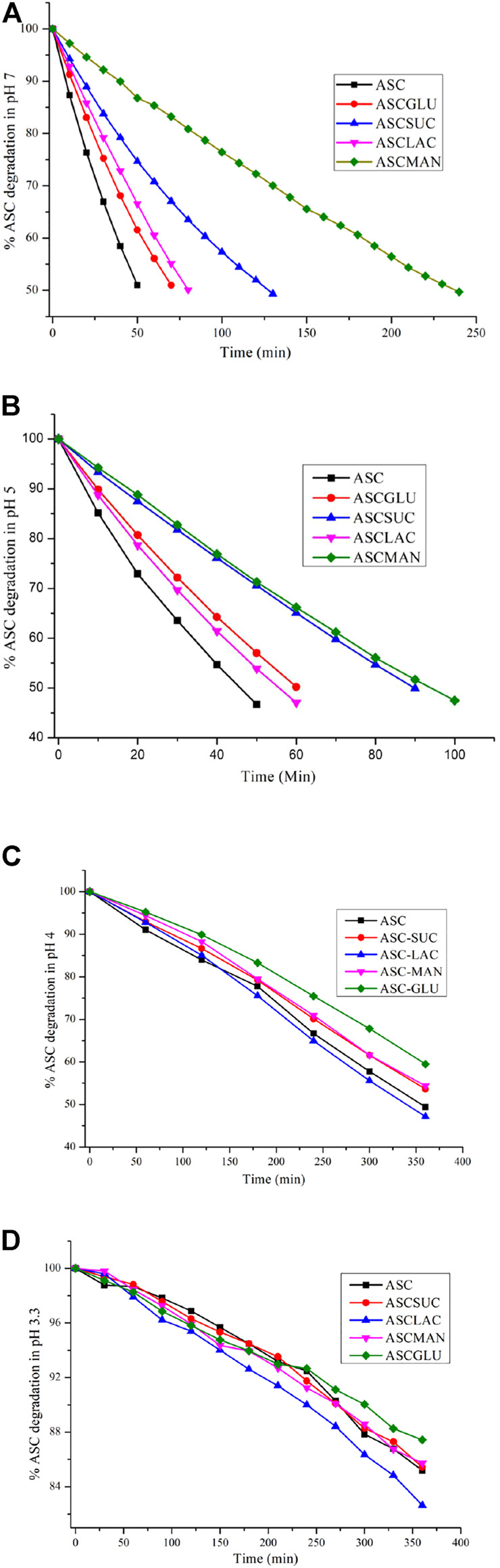
ASC degradation of the sugar eutectics in **(A)** pH 7, **(B)** pH 5, **(C)** pH 4, and **(D)** pH 3.3 acetate buffer medium. Note, UV measurements were recorded at 30 min intervals in pH 4 and 3.3 acetate buffer.

The chemical stability of ASC was carried out using binary eutectics to investigate the effect of the (eutectic) coformers on its shelf-life in different pH media at 25 °C. Note that no additional stabilizing agents were added during the stability experiments. Half-life (T_1/2_) of ASC rapidly decreased from 180 min (pH 4) to 45–50 min (pH 5–7) to 30 min (pH 9) with increasing basicity of the aqueous medium (Figure 3). The acetate (pH 4, 5) and phosphate (pH 7) buffer medium were chosen to compare hydrolytic stability among the binary eutectics. Remarkably, half-life of ASC increased by 2-5-fold by ASC-SUC/Mannitol (MAN) and 1-2-fold by ASC-GLU/LAC eutectics in pH 7 phosphate buffer medium (Figure 4A, Figure S5a, SI). In addition, the ASC cocrystals with nicotinic acid and nicotinamide were subjected to degradation study in pH 7 buffer medium and they degraded much faster compared to pure ASC in pH 7 phosphate buffer medium. The half-life of ASC-nicotinic acid/nicotinamide cocrystals of 15–20 min is much lower compared to that of ASC. The presence of pyridine moiety in nicotinic acid/nicotinamide creates basic environment that may negatively affect the stability aspect of ASC. Among the reported amino acid cocrystals with l-serine, and sarcosine, both remarkably improved shelf-life due to presence of zwitterionic amino acids that play stronger ionic hydrogen bonding interactions with ASC. Of course, the advantage of aqueous stability by ASC-serine/sarcosine is similar to ASC-SUC and ASC-MAN in pH 7 buffer medium. As there were not much stability advantage of the cocrystals compared to the eutectics, we concentrated on sugar eutectics only.

The stability trend of the eutectics was also examined in lower acidic medium like pH 5 acetate buffer medium in which half-life (T_1/2_) of the ASC eutectics was higher than that of ASC (Figure 3B and Figure S5b, SI). The ASC-SUC/MAN eutectics exhibited two-fold higher shelf-life under identical conditions, see Table 2. It was hypothesized that disaccharides like SUC and LAC could improve the aqueous stability of ASC than the monosaccharides (glucose) due to more number of hydroxyl groups involved. In the ASC-LAC (hydrate) eutectics, the presence of one equivalent water molecule in the lattice may spoil the stabilizing advantage of the disaccharides. Unlike cyclic ring sugar moieties, the MAN being a straight chain alcohol shield the lactone and ene-diol moiety of ASC more effectively than GLU. In addition, an equivalent physical mixture of ASC + SUC and ASC + GLU was examined for their hydrolytic stability compared to their ground mixture (eutectics) in pH 5 acetate buffer medium that confirms, eutectics exhibited 1.2–1.3-fold higher half-life compared to their physical counterparts; see Figure S6, SI. Degradation kinetics indicate that the binary eutectics with sucrose and mannitol are the suitable solid forms in terms of enhancing shelf-life of ASC in the weakly acidic (pH 5) and neutral medium (pH 7) compared to ASC or other solid forms. To summarize, the ASC-SUC and ASC-MAN eutectics are promising solid forms that can be used as an injectable formulation (pH 5.6–6.6) with enhanced shelf-life to treat sepsis among patients (Walker et al., 2019).

**TABLE 2:** Half-life period (T_1/2_, min) of ASC binary systems in buffer media^a^ ^,^ ^b^.

ASC Solid Forms (Eutectics)	T_1/2_ (min) in pH 4	T_1/2_ (min) in pH 5	T_1/2_ (min) in pH 7
ASC	180	45	50
ASC-SUC (1:1)	--	90 (x2)	130 (x2.6)
ASC-LAC (1:1)	--	60 (x1.3)	80 (x1.6)
ASC-GLU (1:1)	--	60 (x1.3)	70 (x1.4)
ASC-MAN (1:1)	--	95 (x2.1)	240 (x4.8)
ASC-nicotinic acid^b^	--	--	15
ASC-nicotinamide^b^	--	--	15
ASC-serine^b^	--	95 (x 2.1)	80 (x 1.6)
ASC-sarcosine^b^	--	95 (x 2.1)	85 (x 1.7)

a Number indicates in the parenthesis shows the number of fold improvement.

b Represents ASC, cocrystals reported in the literature (Kavuru et al., 2010; Wang et al., 2016).

Furthermore, degradation study of the binary eutectics was performed in pH 4 acetate buffer medium at 254 nm, which indicated that stabilizing advantage of the ASC-SUC/MAN decreased and that of the ASC-GLU enhanced to some extent, see Figure 4C. After 6 h of degradation studies, ASC remained at 60% in ASC-GLU and 53–54% in ASC-SUC/MAN binary eutectic compared to 50% in native ASC. Surprisingly, stabilizing effect of the ASC-LAC (hydrate) system vanished. The presence of one equivalent water molecule in the lattice of the ASC-LAC (hydrate) eutectic could destabilize ASC effectively even in the presence of the disaccharide (LAC). Stabilizing effect of the ASC-SUC eutectics was nullified in the acidic medium due to transformation of sucrose (disaccharides) to glucose and fructose (monosaccharides). Among the carbohydrates, the fructose showed higher degradation effect on ASC that could play a negative role to overcome the positive stabilizing effect of glucose (Tikekar et al., 2011). On a similar note, mannitol also transformed to fructose and decreased shelf-life of ASC in the acidic medium.

To examine the effect of ASC binary eutectics in increasing its shelf-life in the model juice medium (pH 3.2–3.5), degradation experiment was examined in acetate buffer medium (pH 3.3) at 245 nm. As expected, the ASC degradation was very slow in the acidic medium, which confirmed only ∼15% ASC degraded after 6 h. All the sugar eutectics exhibited similar degradation behavior in the lower pH medium, see Figure 4D. No stabilizing effect of ASC-SUC/MAN//GLU eutectics was found in the model apple juice, i.e., malic acid solution (0.5% w/v). This suggests that malic acid (pKa 3.4) is strongly acidic enough to hydrolyze sucrose to glucose and fructose (*via* acidic hydrolysis), similar to acetate buffer (of pH 3.3).

Passing UV radiation to eliminate microbial levels in fruit juice/food products is an established technique compared to thermal treatment to avoid degradation. Effect of ASC (∼final conc. 125 mg/L) and ASC-SUC/MAN eutectics in malic acid/acetate buffer medium (pH 3.3) under continuous UV photodegradation was found to be negligible owing to the rapid saccharide radical formation (Kubota et al., 1978), which increased faster degradation of ASC.

### Binding Energy Calculations

**FIGURE 5:**
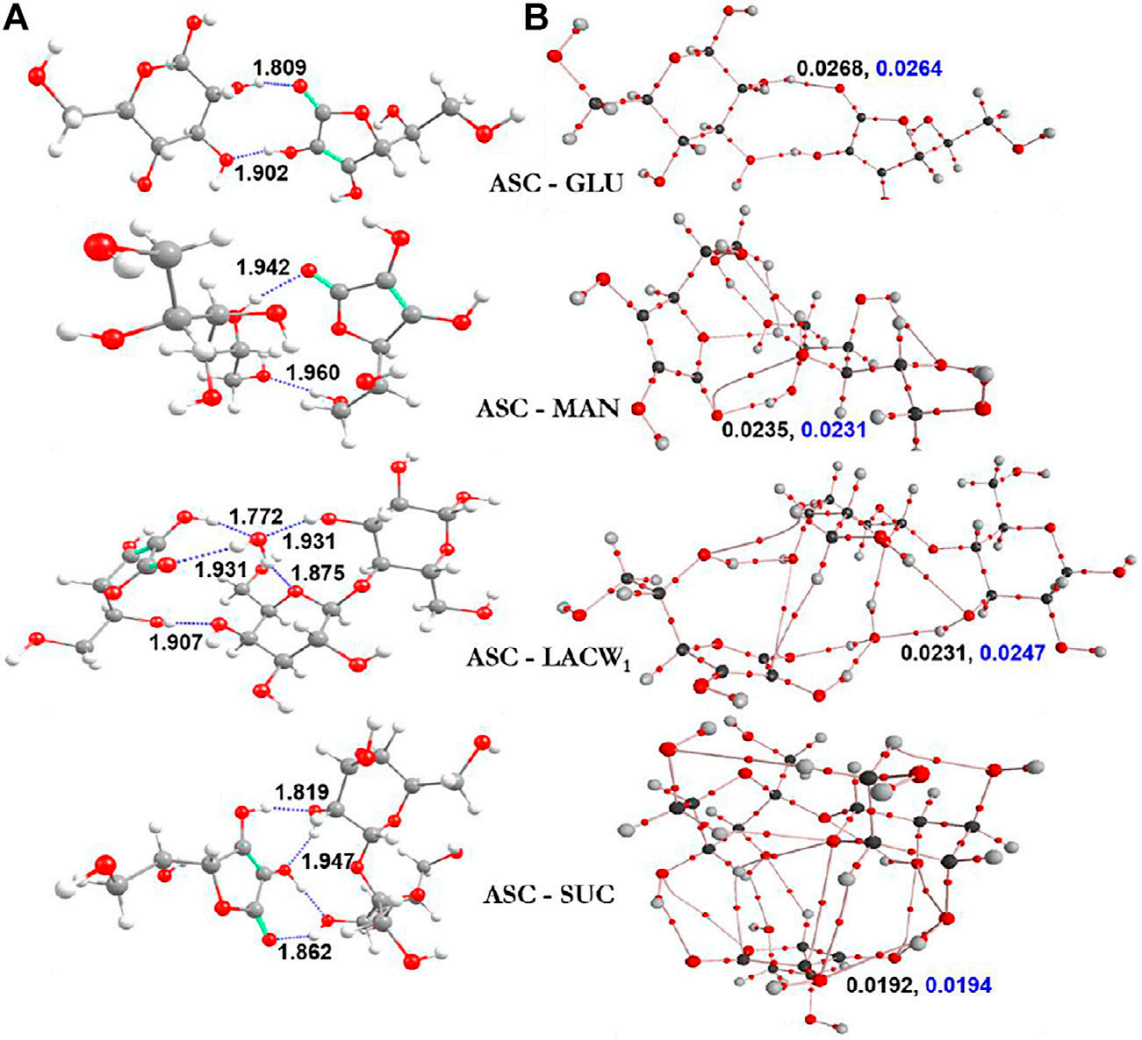
Ascorbic acid with sugars of **(A)** the most stable optimized geometries (along with distances Å) and **(B)** electron [ρ(r_c_)] and Laplacian [∇^2^ρ(r_c_)] density values are displayed in black and blue colors, respectively.

The optimized geometries of ASC with selected sugars (glucose, sucrose, lactose monohydrate, and mannitol) are demonstrated in Figure 5 **.** The corresponding BEs of the most stable geometries in both gas and aqueous phase are summarized in Table 3 **.** Computation indicates that the number of hydrogen bonding interactions is responsible for the stability of the eutectic complexes. The complex stability and the magnitude of complex strength are in the order of ASC‒GLU < ASC‒MAN < ASC‒LACW_1_ < ASC‒SUC in the gas phase. The same complexes in the aqueous phase (i.e., water) also predict the similar trend. The disaccharides (such as sucrose and lactose monohydrate) strongly interact with ASC due to the formation of additional H-bonded interactions between sugar and ASC, whereas the monosaccharides (such as GLU and MAN) form a lesser number of O‒H^**…**^O‒H and O‒H^**…**^O=C H-bonding interactions. Moreover, OH groups of the sugars constitute H-bonding with the carbonyl group of ASC. This confirms the stability of the ASC binary complex, which depends on the selection of sugars in the study and in this case preferably with sucrose.

**TABLE 3:** The binding energies (kcal/mol) of ASC eutectics in gas phase and water phase.

	Gas Phase		PCM (Aqueous Phase)	
Complexes	M05-2X	M05-2X + D3	M05-2X	M05-2X + D3
ASC-GLU	−18.32	−19.33	−17.16	−18.12
ASC-MAN	−18.28	−20.8	−17.84	−20.36
ASC-SUC	−25.44	−27.83	−24.96	−27.27
ASC-LACW_1_	−20.95	−23.53	−19.89	−23.57

The role of water molecule at the vicinity of sugar is very important to study the stability order of the clusters. The lactose showed stronger interactions with ASC (‒27.34 kcal/mol), whereas in the presence of water molecule the interaction energy slightly decreased (‒20.95 kcal/mol). Lactose monohydrate system was taken for the binding interaction with ASC as the single water molecule in the lattice can alter the stability and the molecular properties. This is due to the electrostatic behavior of the complex (ASC-LACW_1_), which is completely different from the remaining other clusters. This arises from the interfacial interactions between sugar and ASC, which are altered by the incoming water molecules. This is due to the formation of H-bond pattern at central water molecule, which acts as an AADD model (i.e., double acceptor double donor) for LACW_1_ system. On the other hand, GLU, MAN, and SUC form AD-type of H-bond interaction. This confirms that the existence of water molecule acts as a bridge between ASC and LAC, which significantly reduce the complex stability. This is due to the formation of double donor-type hydrogen bonded water molecule. A similar kind of finding was also observed in earlier report on hydrated systems (Prakash et al., 2011). The presence of one water molecule partially disturbs the stability gained by the disaccharide lactose. Computational results reveal that disaccharides (SUC and LACW_1_) exhibit stronger binding affinity with ASC than their monosaccharide counterparts.

To gain more insight into the stability of clusters, individual interaction strength in various complexes was quantified through AIM topography analysis. The bond critical points (BCPs) of electron density and Laplacian values are helpful to understand the nature of interactions in the clusters, see Figure 5B. It was observed from our calculations that there are several noncovalent interactions in the binary complexes. From the BEs calculations, SUC and LAC exhibit strong noncovalent interactions with ASC, which improved the aqueous stability of the corresponding binary eutectics. To conclude, BE calculation of the ASC eutectics partially supports the experimental aqueous stability data as the ASC-MAN offers the suitable solid form with the highest shelf-life of ASC.

## Conclusion

A few binary eutectics of ASC with sugars were prepared using wet granulation method and their stabilizing behavior against hydrolytic degradation was examined. Eutectic compositions between ASC and sugars are formed due to non-compatible heterosynthons and analogous geometry of the counter molecules. All the binary eutectics were non-hygroscopic up to 80% RH at ambient conditions and can be stored for long time. Among the binary complexes, the sucrose and mannitol eutectics enhanced shelf-life of ASC by 2–5-fold in pH 5 and 7 buffer medium, although their stabilizing effect was reduced with decreasing pH of the medium. Binding energy calculations of the binary eutectic mixtures in the aqueous phase confirm the improved stabilizing effect of disaccharides than monosaccharides due to more number of non-covalent interactions. It is anticipated that the enhanced aqueous stability of ASC using eutectics in the minute concentration will promote an equivalent effect in the higher concentrated ASC containing aqueous phase. To conclude, the application of the ASC-GLU eutectics as vitamin C supplement in fruit juices (acidic pH) and ASC-MAN as injectable formulation (pH 5.6–6.6) will be promising to use. The search for suitable coformers using the crystal engineering approach to enhance the aqueous stability of ASC will be continued.

## Data Availability

The original contributions presented in the study are included in the article/Supplementary Material, further inquiries can be directed to the corresponding authors.
