# Supplementary material for: Design of Ascorbic Acid Eutectic Mixtures With Sugars to Inhibit Oxidative Degradation
Source: Front Chem. 2022 May 9;10:754269. doi: 10.3389/fchem.2022.754269 (PMC9125031; doi:10.3389/fchem.2022.754269)
Supplement: Supplementary file 1 [file DataSheet1.pdf]

Supporting information

Design of Ascorbic Acid Eutectic Mixtures with Sugars to Inhibit Oxidative Degradation

Vasanthi Palanisamy,[a] Palash Sanphui,*[a] Kandhan Palanisamy,[a] Muthuramalingam Prakash[a] and Arvind Kumar Bansal*[b]

[a]Department of Chemistry, Faculty of Engineering and Technology, SRM Institute of Science and Technology, SRM Nagar, Kattankulathur, Chennai-603203, India. Email: palashi@srmist.edu.in

[b]Department of Pharmaceutics, National Institute of Pharmaceutical Education and Research, S.A.S. Nagar, Mohali-160062, Punjab, India. Email: akbansal@niper.ac.in

Table of Contents

1. PXRD overlay of ASC-sugar eutectics with ASC and sugars……………………….Figure S1

2. Vibrational frequencies comparison of ASC-sugar eutectics with ASC and sugars…Figure S2

3. DSC/TGA plots of ASC-LAC (hyd)…………………………………………………Figure S3

4. FESEM images of ASC-sugar eutectics………………………………………………..Figure S4

5. Aqueous stability data and UV plots……………………………………………………Figure S5

6. Degradation comparison of ASC-sugar eutectics and ASC + sugar (physical mixture)...Figure S6

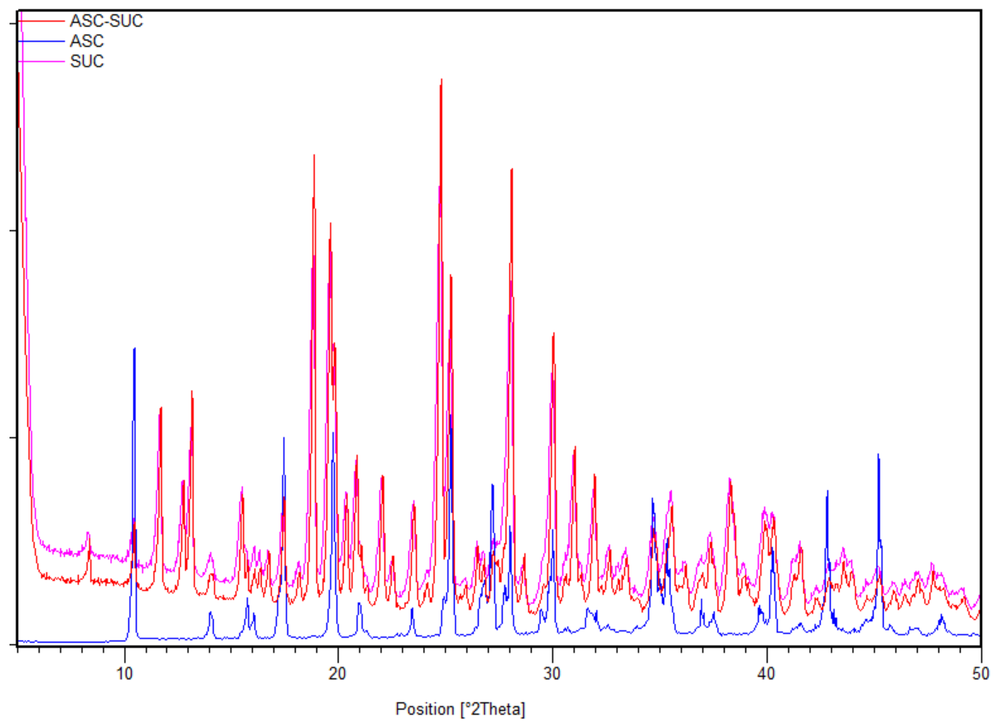

ASC-SUC
ASC
SUC
10
20
30
40
50
Position [°2Theta]

(a)

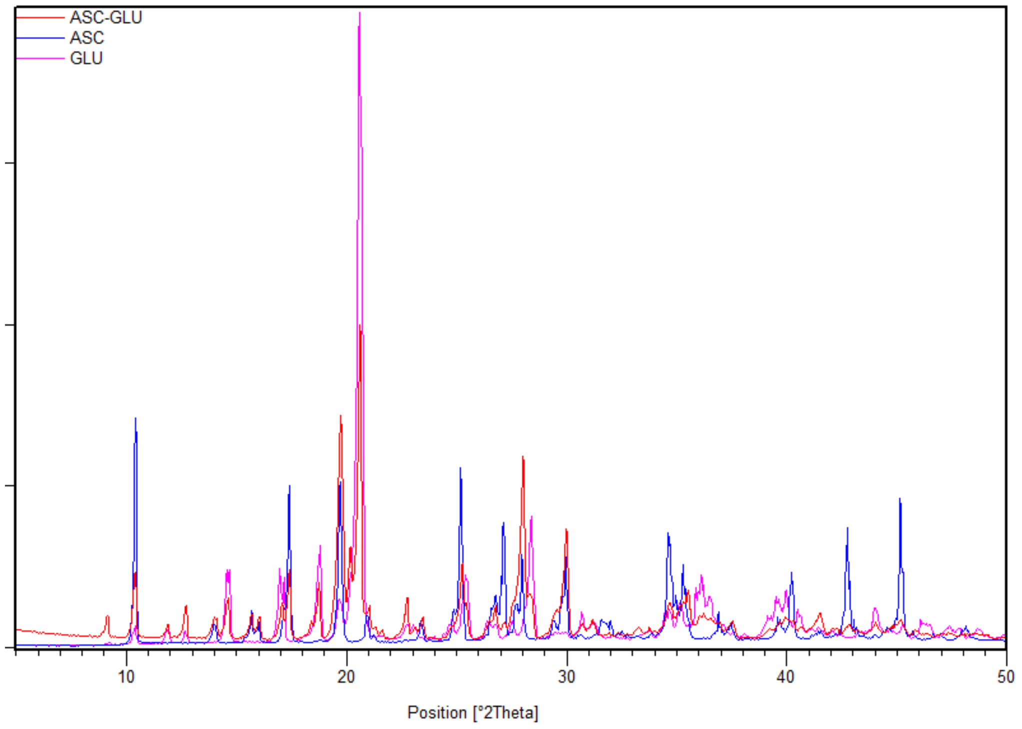

ASC-GLU
ASC
GLU
10
20
30
40
50
Position [°2Theta]

(b)

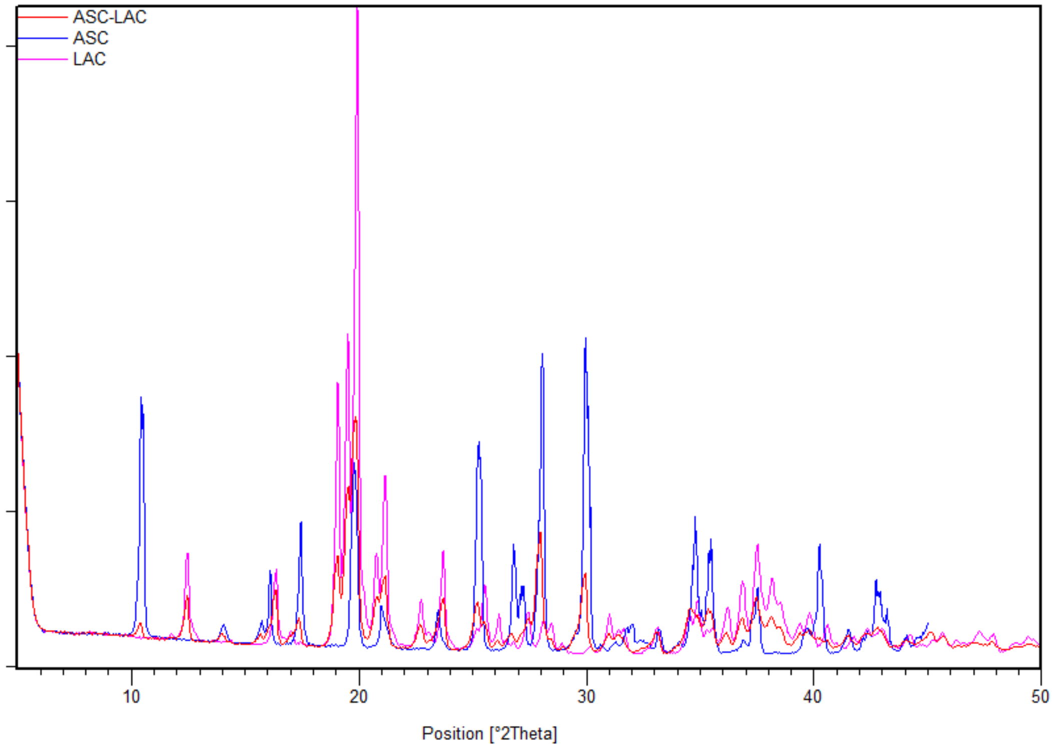
ASC-LAC
ASC
LAC
10
20
30
40
50
Position [°2Theta]

(c)

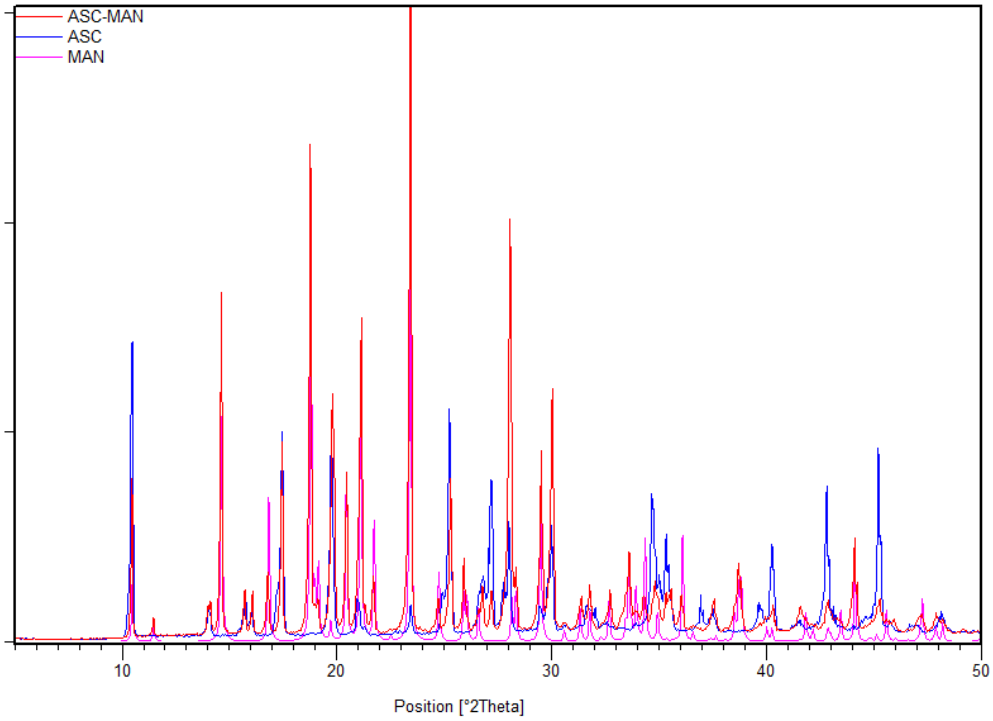
ASC-MAN
ASC
MAN
10
20
30
40
50
Position [°2Theta]

(d)

FIGURE S1. PXRD overlay of (a) ASC-SUC, (b) ASC-GLU, (c) ASC-LAC (hydrate) and (d) ASC-MAN binary eutectics with ASC and sugar coformers.

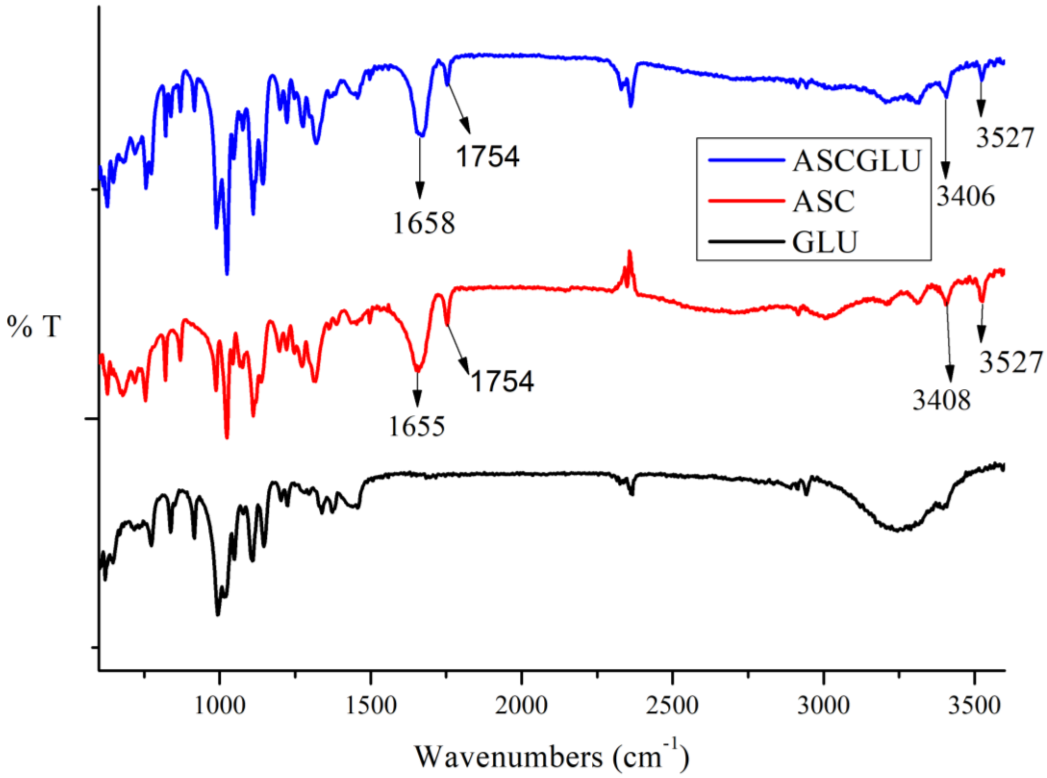

(a)

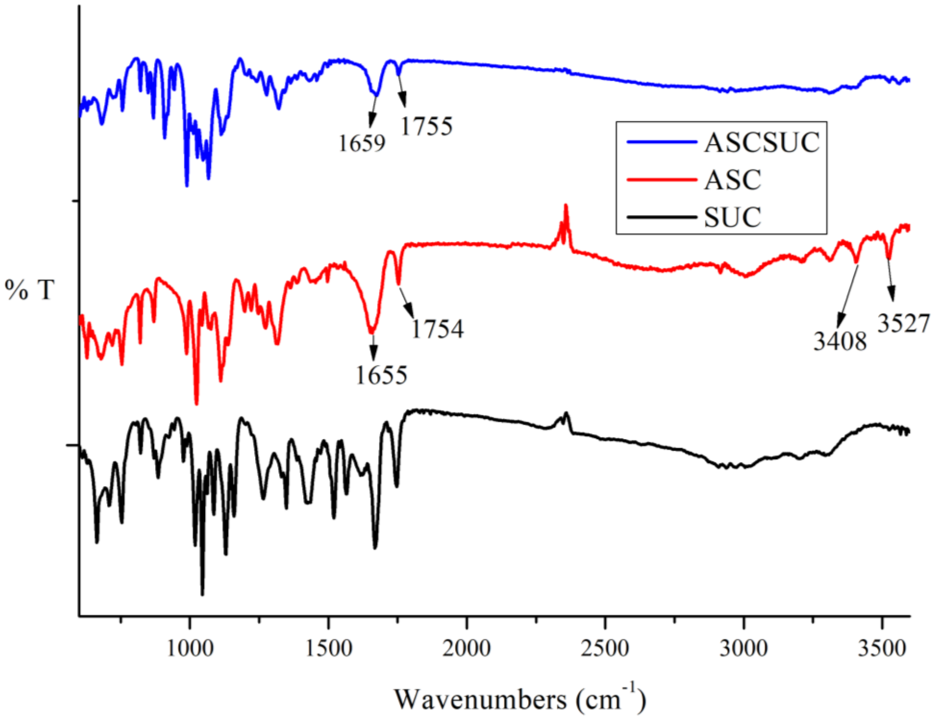

(b)

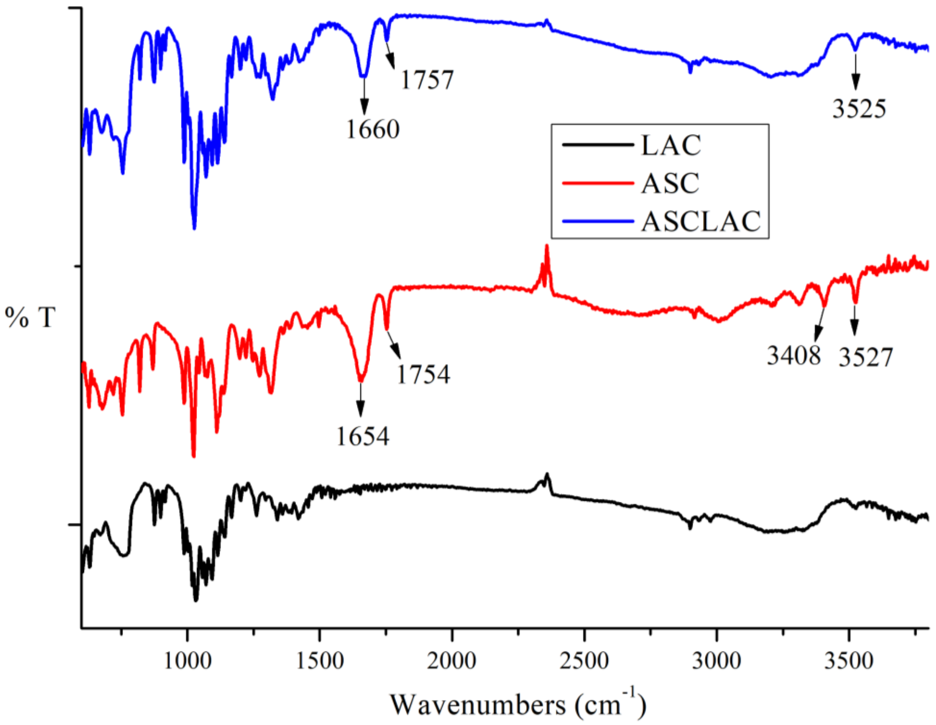

(c)

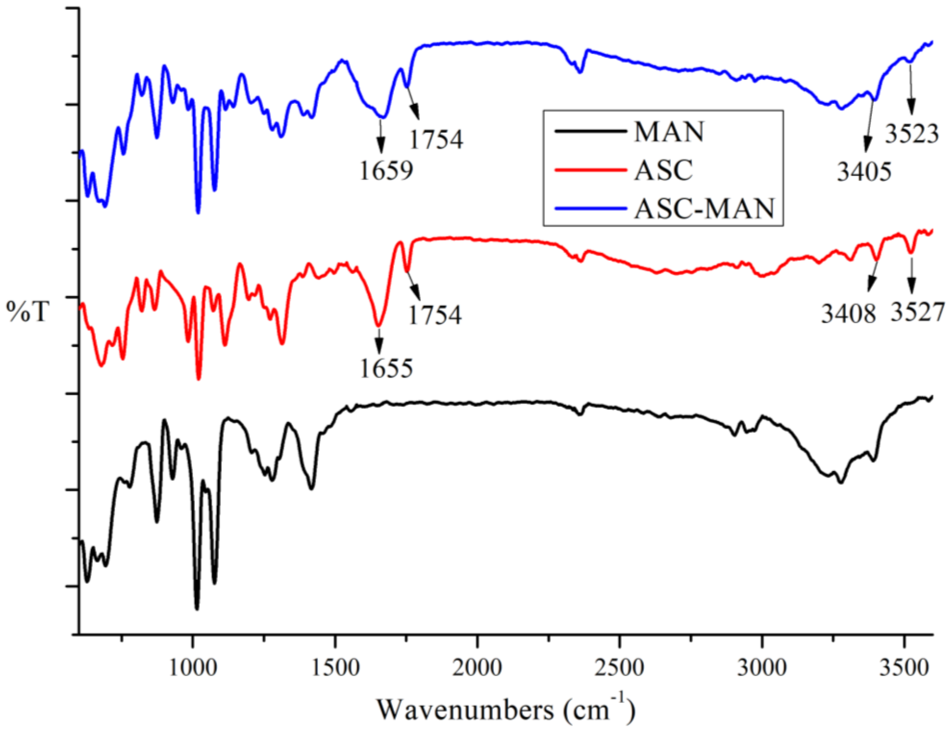

(d)

FIGURE S2. Vibrational frequencies comparison of (a) ASC-GLU, (b) ASC-SUC, (c) ASC-LAC (hydrate) and (d) ASC-MAN binary eutectics with ASC and sugar coformers.

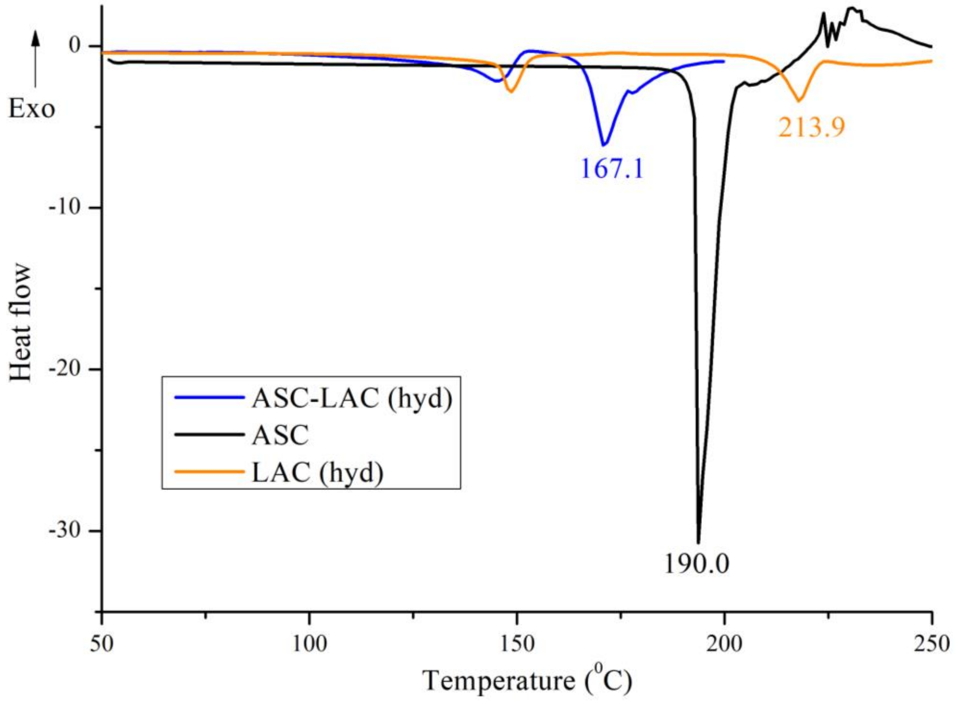

(a)

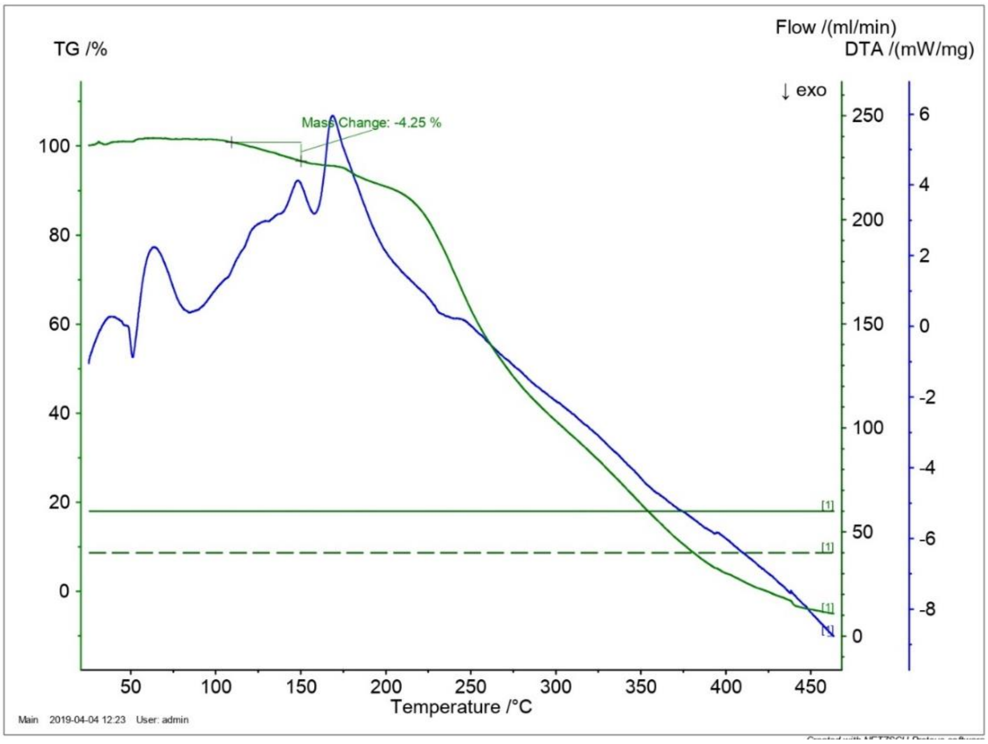

(b)

FIGURE S3. (a) DSC comparison of ASC-LAC (eutectic), ASC and LAC (sugar) that indicates the dehydration pattern of the binary eutectic and coformer LAC are different. (b) TGA data of ASC-

LAC indicates that weight loss due to dehydration started at ~115-120 ^{o}C, followed by gradual weight loss up to melting and rapid loss thereafter.

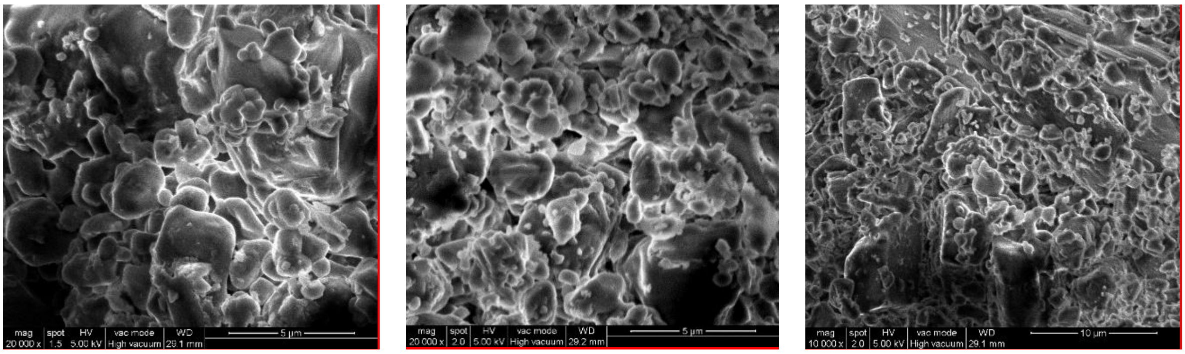

ASC-SUC ASC-LAC ASC-MAN

FIGURE S4. FESEM images of ASC-sugar eutectics (powder), which indicate irregular morphology.

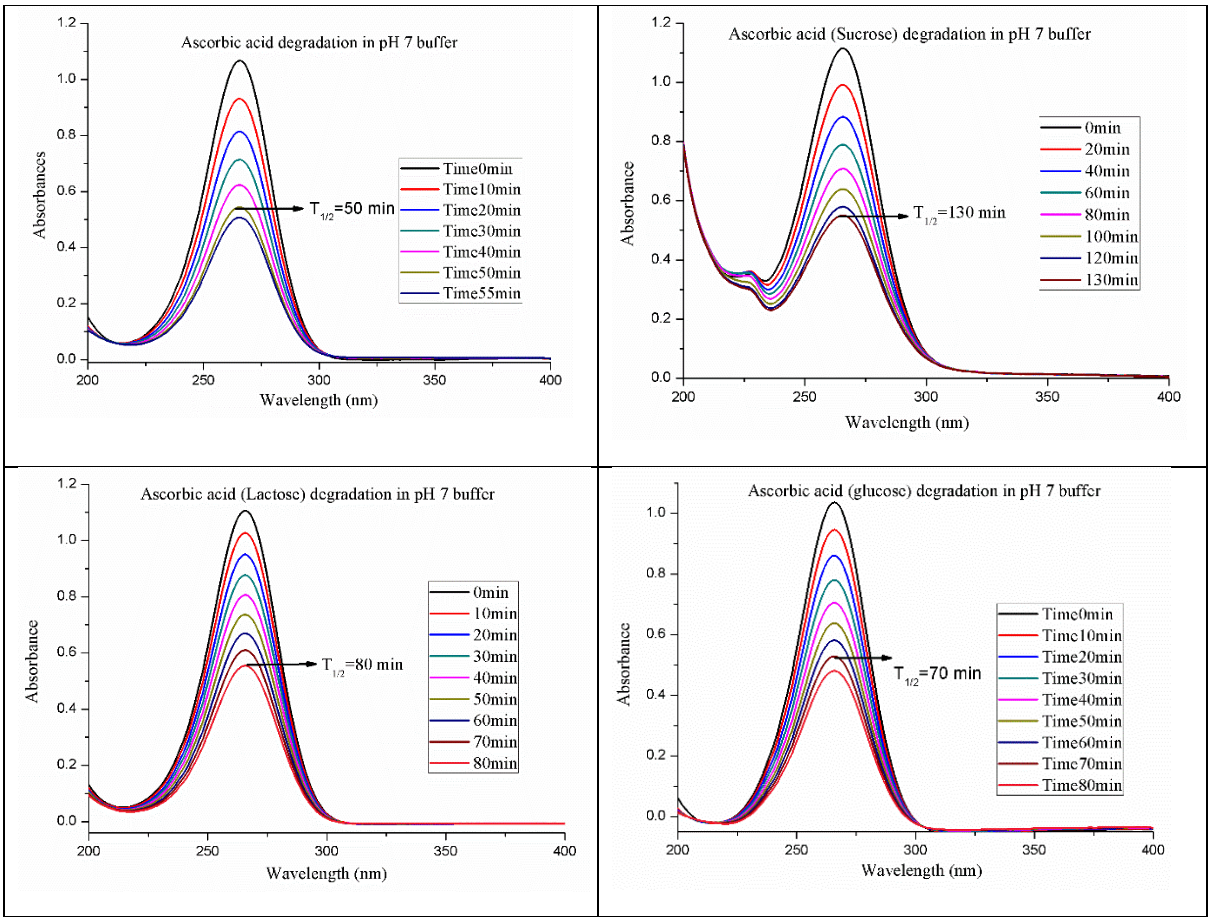

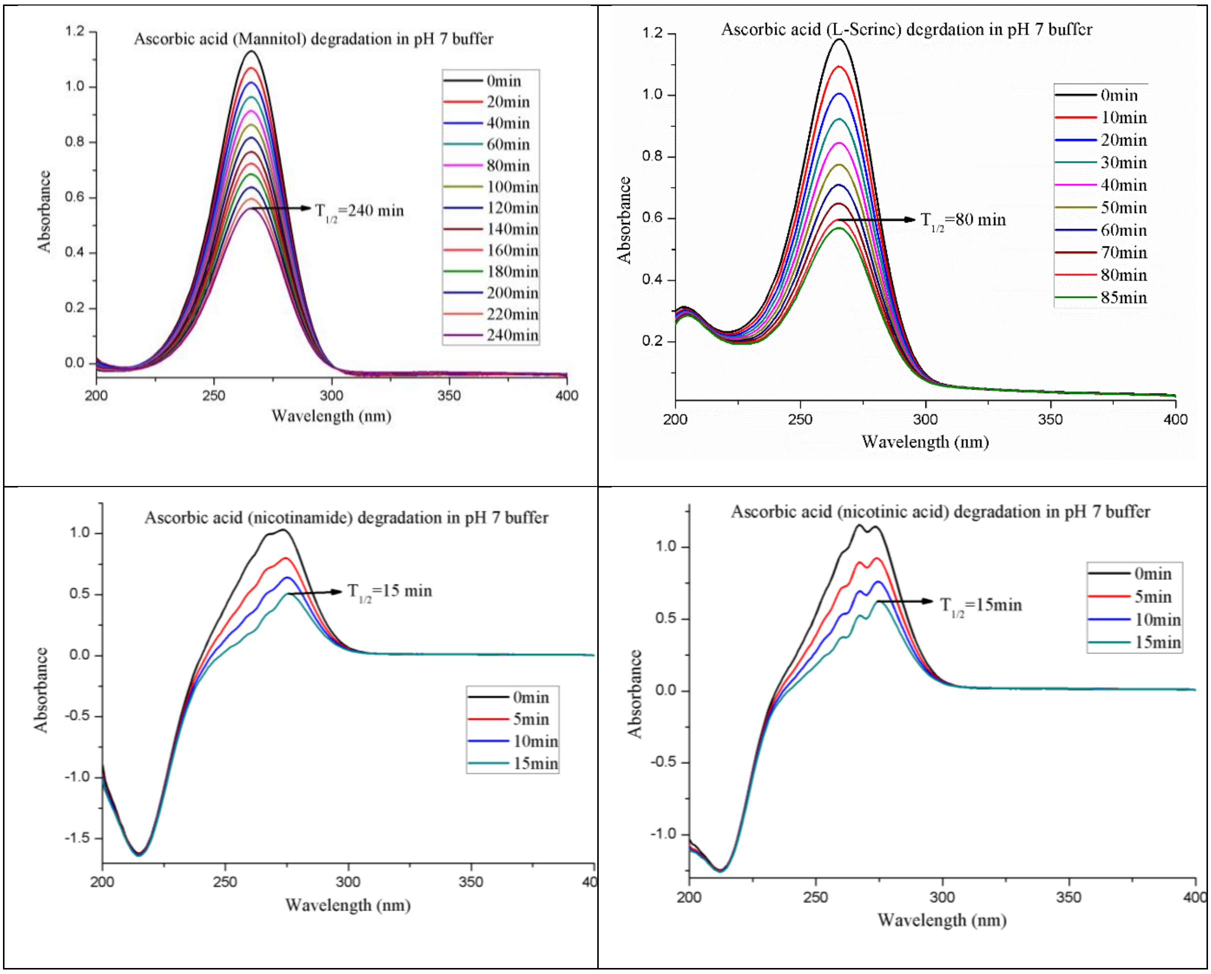

Ascorbic acid (Mannitol) degradation in pH 7 buffer
Absorbance
Wavelength (nm)
T1/2=240 min
0min
20min
40min
60min
80min
100min
120min
140min
160min
180min
200min
220min
240min
Ascorbic acid (L-Serinc) degrdation in pH 7 buffer
Absorbance
Wavelength (nm)
T1/2=80 min
0min
10min
20min
30min
40min
50min
60min
70min
80min
85min
Ascorbic acid (nicotinamide) degradation in pH 7 buffer
Absorbance
Wavelength (nm)
T1/2=15 min
0min
5min
10min
15min
Ascorbic acid (nicotinic acid) degradation in pH 7 buffer
Absorbance
Wavelength (nm)
T1/2=15min
0min
5min
10min
15min

(a)

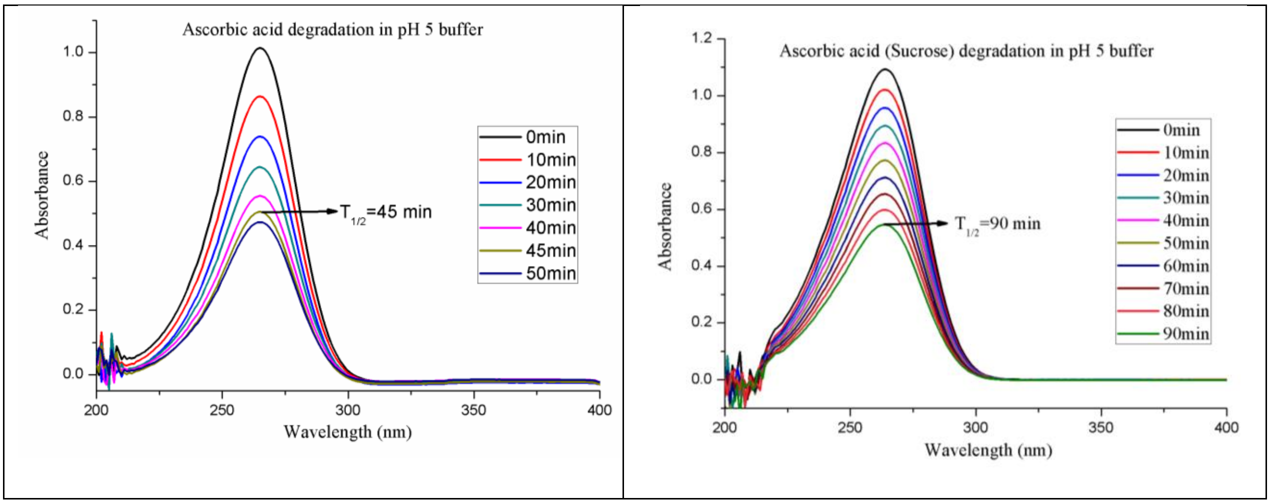

Ascorbic acid degradation in pH 5 buffer
Absorbance
Wavelength (nm)
T1/2=45 min
0min
10min
20min
30min
40min
45min
50min
Ascorbic acid (Sucrose) degradation in pH 5 buffer
Absorbance
Wavelength (nm)
T1/2=90 min
0min
10min
20min
30min
40min
50min
60min
70min
80min
90min

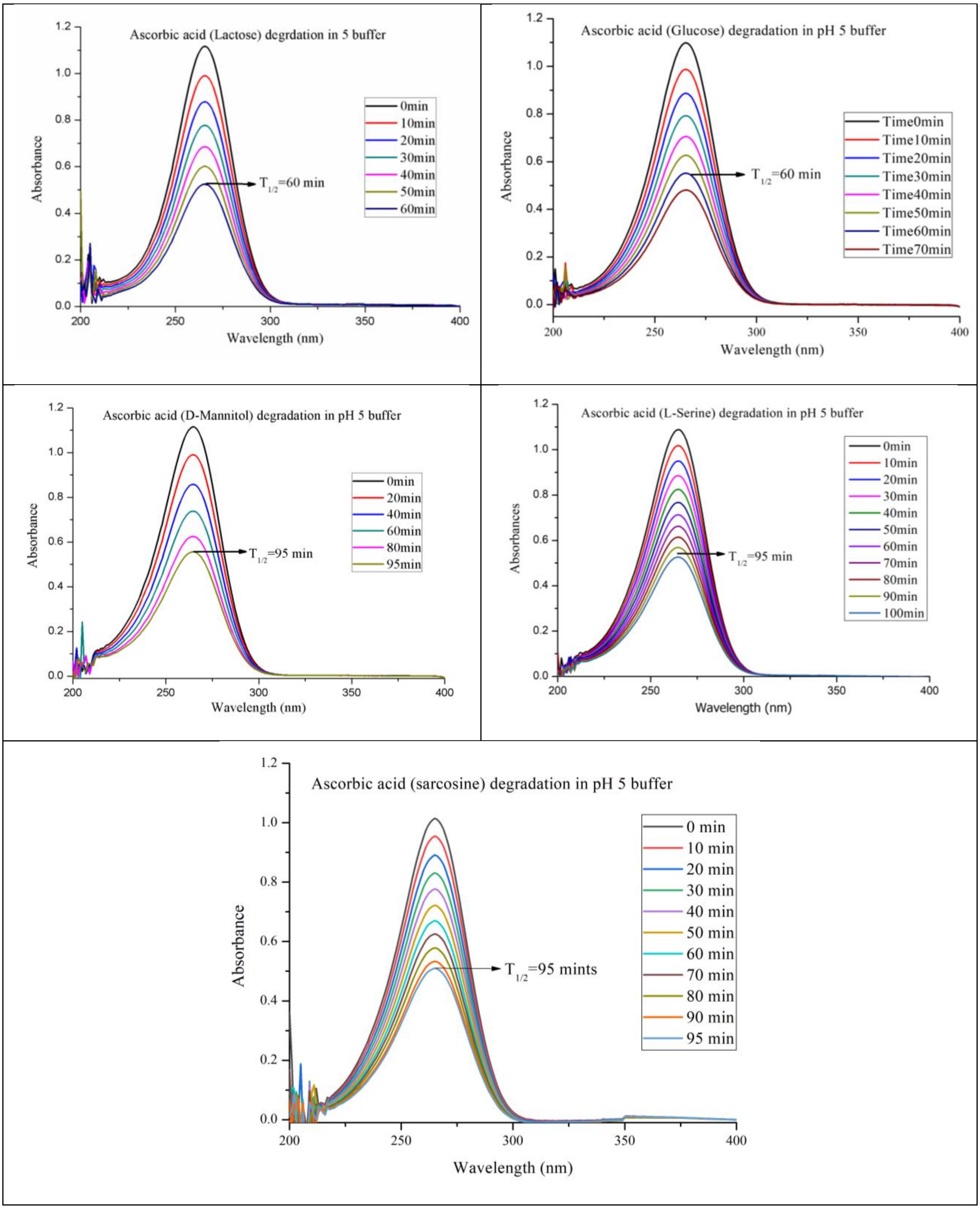

(b)

FIGURE S5. ASC degradation study of ASC-sugar eutectics and ASC-serine/sarcosine/nicotinic acid/nicotinamide cocrystals in (a) pH 7 (phosphate) and (b) pH 5 (acetate) buffer medium at 25 ^{O}C.

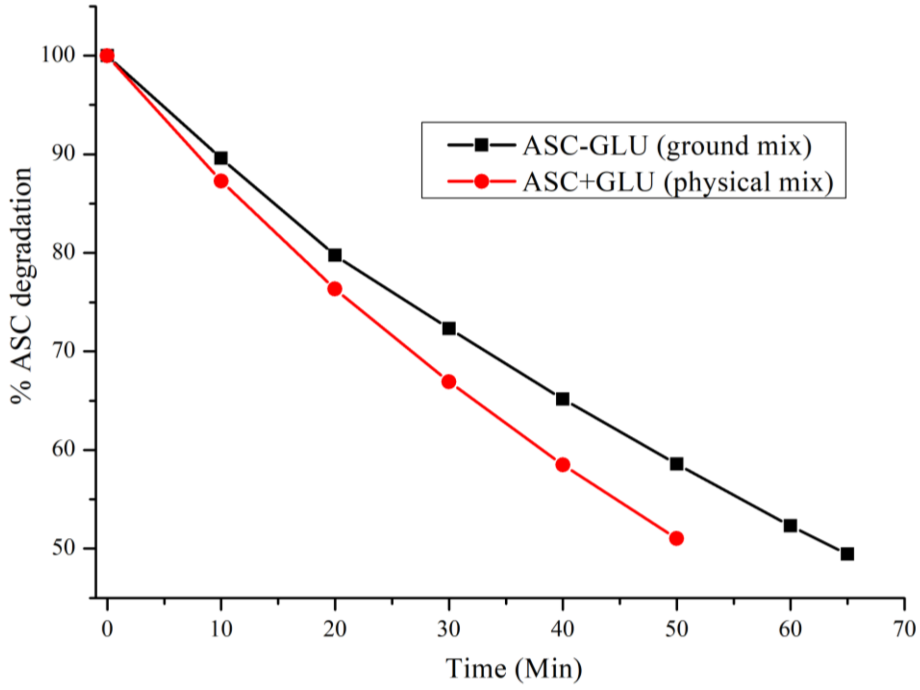

(a)

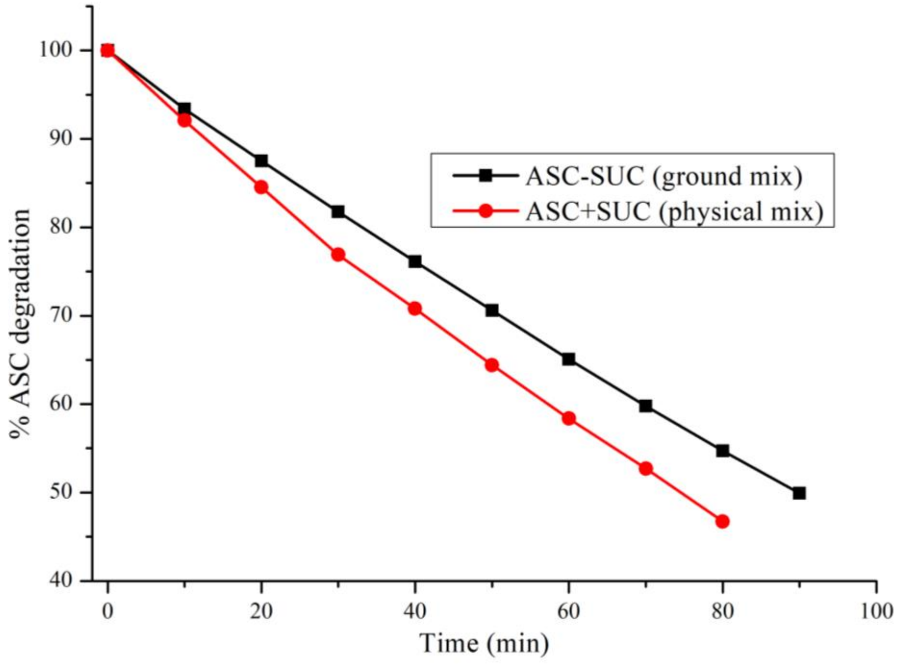

(b)

FIGURE S6. ASC degradation comparison in between (a) ASC-GLU (ground vs physical mixture) and (b) ASC-SUC in pH 5 acetate buffer medium, which indicate equimolar ground (eutectic) mixture improved shelf-life of ASC than their physical mixtures.
