# Supplementary material for: Design of Ascorbic Acid Eutectic Mixtures With Sugars to Inhibit Oxidative Degradation
Source: Front Chem. 2022 May 9;10:754269. doi: 10.3389/fchem.2022.754269 (PMC9125031; doi:10.3389/fchem.2022.754269)
Supplement: Supplementary file 2 [file Image1.pdf]

Graphical Abstract

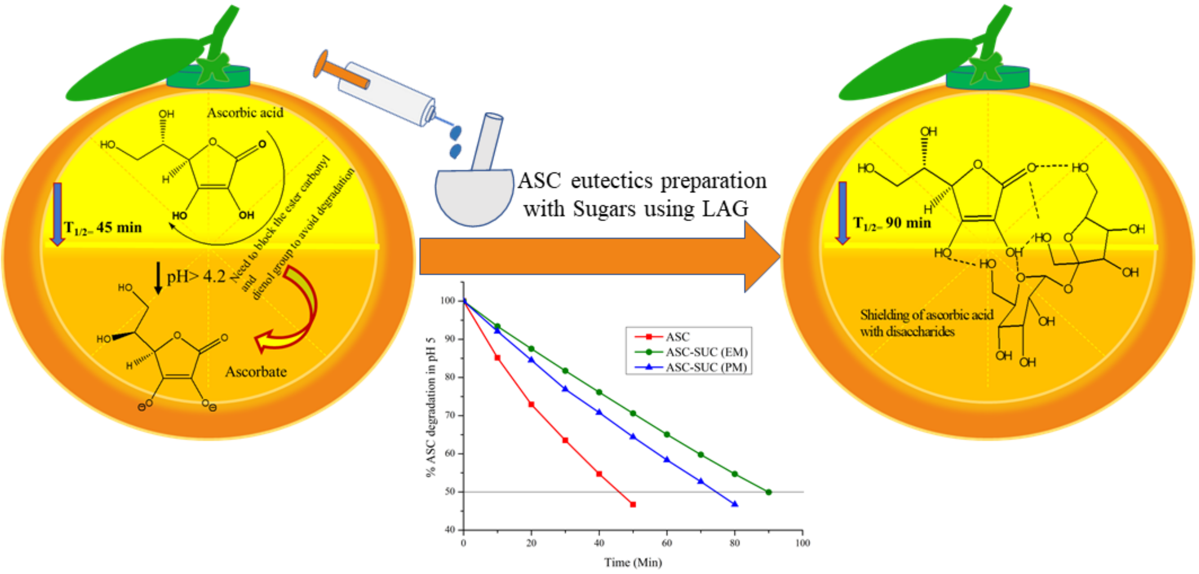

Ascorbic acid binary eutectics with mono/disaccharides increased 2-5 fold half-life in pH 5 and 7 buffer medium that promotes importance of eutectics in modulating chemical stability.
